# Selective plasmon-driven catalysis for para-nitroaniline in aqueous environments

**DOI:** 10.1038/srep20458

**Published:** 2016-02-09

**Authors:** Lin Cui, Peijie Wang, Yuanzuo Li, Mengtao Sun

**Affiliations:** 1 The Beijing Key Laboratory for Nano-Photonics and Nano-Structure, Department of Physics, Capital Normal University, Beijing, 100048, People’s Republic of China; 2 Beijing National Laboratory for Condensed Matter Physics, Institute of Physics, Chinese Academy of Sciences, Beijing, 100190, People’s Republic of China; 3 College of Science, Northeast Forestry University, Harbin, 150040, People’s Republic of China

## Abstract

The plasmon-driven oxidation of amine (−NH_2_) groups and the reduction of nitro (−NO_2_) groups on a nanostructured metal surface in an aqueous environment have been reported experimentally and theoretically. The question of which process occurs first in the aqueous environment is an interesting question in the field of plasmon-related photochemistry. Para-nitroaniline (PNA), with both nitro (−NO_2_) and amine (−NH_2_) groups, is the best candidate for studying the priority of the plasmon-driven oxidation and the reduction reactions in an aqueous environment. Using surface-enhanced Raman scattering (SERS) spectroscopy, our experimental results and theoretical simulations reveal that PNA is selectively catalyzed to 4,4′-diaminoazobenzene (DAAB) through the plasmon-assisted dimerization of the nitro (−NO_2_) group into an azo group in an aqueous environment. This indicates that the plasmon-driven reduction of the nitro (−NO_2_) group clearly occurs before the oxidation of the amine (−NH_2_) group in an aqueous environment. The plasmon-driven reduction of PNA to DAAB is a selective surface catalytic reduced reaction in aqueous environment.

Since the first report of the plasmon-driven oxidation reaction in which para-aminothiophenol (PATP, see Fig. 1a) is catalyzed to p,p′-dimercaptoazobenzene (DMAB, see Fig. 1b) in 2010^1^,^2^, the realization of plasmon-driven chemical reactions has been one of most important advances in the field of nanoplasmonics^3^,^4^,^5^,^6^,^7^,^8^,^9^,^10^,^11^,^12^,^13^,^14^,^15^,^16^, which is also called plasmon chemistry^8^. Hot electrons^17^,^18^,^19^, which generated from plasmon decay, play an important role in the plasmon-driven catalytic reactions. In the plasmon-driven oxidation reactions, the neutral potential energy surface can become negatively charged as hot electrons temporarily attach to molecules, and then decrease the reaction barrier. Furthermore, the kinetic energy of hot electrons can be efficiently transferred to molecules to provide energy for the reduction reaction. In addition to the above advantages of plasmon-driven oxidation reactions, hot electrons can serve as the required electron source in plasmon-driven reduction reactions.

It has been reported that DMAB can be catalytically produced from PATP through a plasmon-driven oxidation reaction^1^,^2^ or from 4-nitrobenzenethiol (4NBT, see Fig. 1c) through a plasmon-driven reduction reaction^5^,^6^. Several recent review papers are available on this topic^20^,^21^,^22^,^23^,^24^. However, it is not clear what role surface plasmons play in the priority of these catalytic reactions. Para-nitroaniline (PNA, see Fig. 1d) contains both nitro (−NO_2_) and amine (−NH_2_) groups, making it the best candidate for studying the priority of the plasmon-driven oxidation and the reduction reactions. Three new molecules (see Fig. 1e–g) could be produced from PNA according to the priority of plasmon-driven chemical reactions. If plasmon-driven oxidation reactions occur first, then 4,4′-dinitroazobenzene (DNAB) can be produced. However, if plasmon-driven reduction reactions occur first, 4,4′-diaminoazobenzene (DAAB) can be produced. The third possibility is the occurrence of simultaneous oxidation and reduction reactions that produce 4-nitro-4′-aminoazobenzene (NAAB). This question is a highly interesting topic in plasmon-driven catalytic reaction in the aqueous environment.

In this reports, the plasmon-driven chemical reaction of PNA in an aqueous environment was investigated experimentally using electrochemical surface-enhanced Raman scattering (SERS) spectroscopy, and simulated with density functional theory (DFT). Our results revealed that PNA was selectively reduced to DAAB with the help of surface plasmon in the aqueous environment.

## Results and Discussion

**Figure 2:**
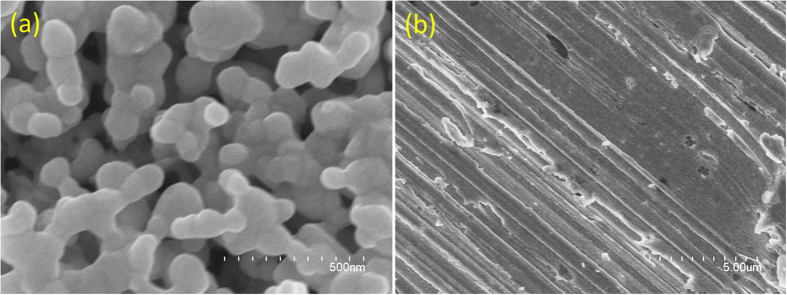
(**a**) The SEM image of the roughened Ag substrate, and (**b**) the SEM image of the less roughened Ag substrate.

The roughened Ag electrodes and less roughened Ag electrodes can be observed on the nanoscale in the SEM image in Fig. 2(a,b), respectively. Due to the larger hot sites in the roughened Ag electrodes, there are stronger electromagnetic enhancements for the detecting Raman signals for the catalytic reactions.

**Figure 3:**
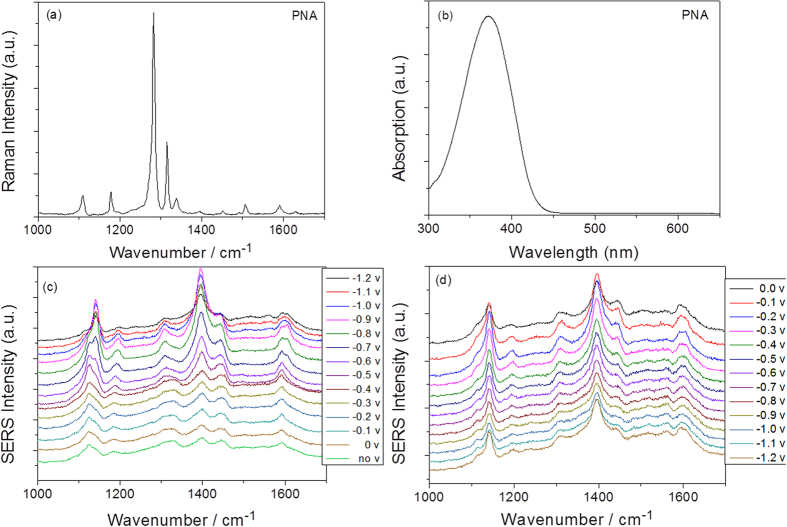
(**a**) Molecular Raman spectrum of PNA, (**b**) Absorption spectrum of PNA, (**c**) potential dependent electrochemical SERS spectra of PNA from 0 V to −1.2 V, and (**d**) potential dependent electrochemical SERS spectra of PNA from −1.2 V back to 0 V again.

The Raman spectrum of the PNA powder, which was measured as a reference, is shown in Fig. 3(a). The absorption spectrum of PNA can be seen from Fig. 3(b). Furthermore, we also measured the electrochemical SERS spectra of PNA on the roughened Ag substrate in the aqueous environment (see Fig. 3(c,d)). From Fig. 3(b), it can be concluded that SERS spectra excited at 532 nm are normal Raman spectra. Figure 3(c) shows potential-dependent SERS spectra of PNA in an aqueous environment, where the potential varies from 0 V to −1.2 V. When comparing Fig. 3(a,c), the SERS profiles are significantly different. Plasmon-driven chemical reactions appeared to occur through the amine (−NH_2_) and/or nitro (−NO_2_) groups of PNA in the aqueous environment during the spectral measurements.

**Figure 4:**
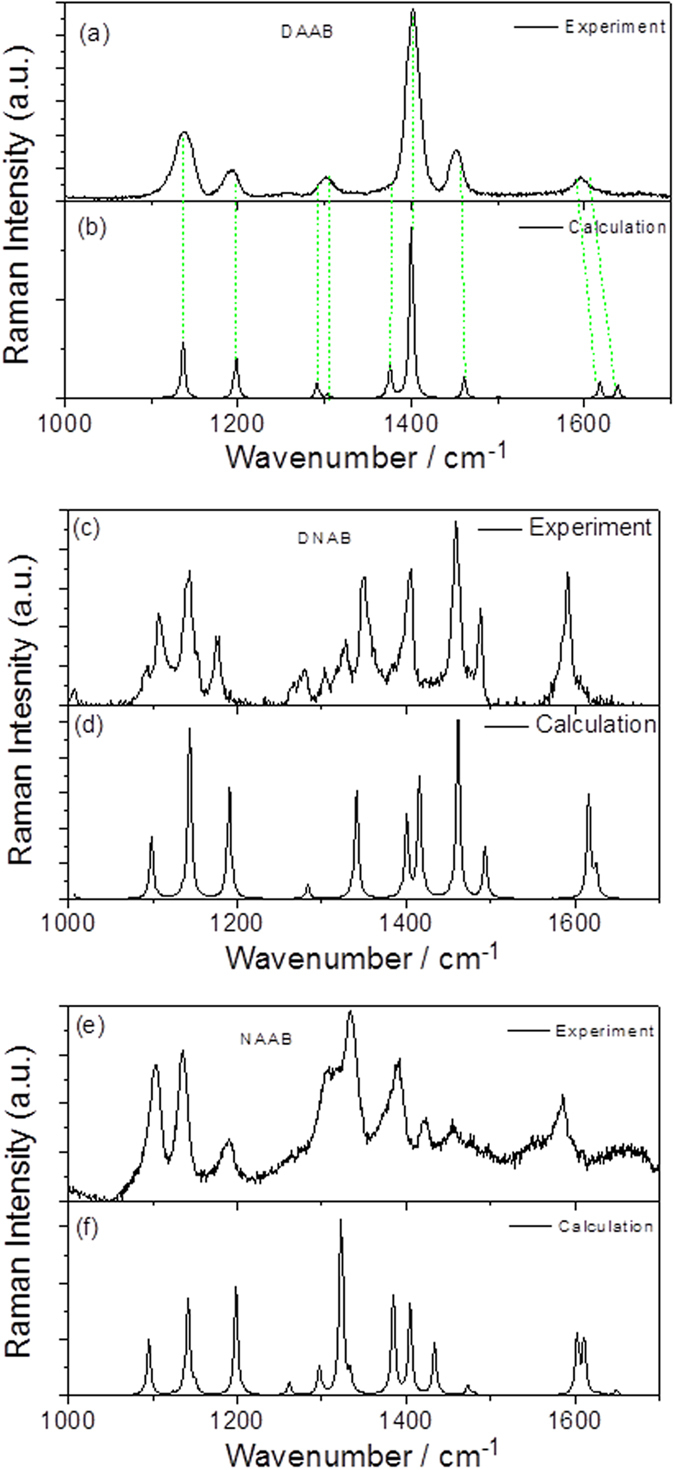
Molecular Raman spectra excited at 532 nm. (**a**,**b**) experimental and simulated Raman spectra of DAAB, (**c**,**d**) experimental and simulated Raman spectra of DNAB, and (**e**,**f**) experimental and simulated Raman spectra of NAAB.

**Figure 5:**
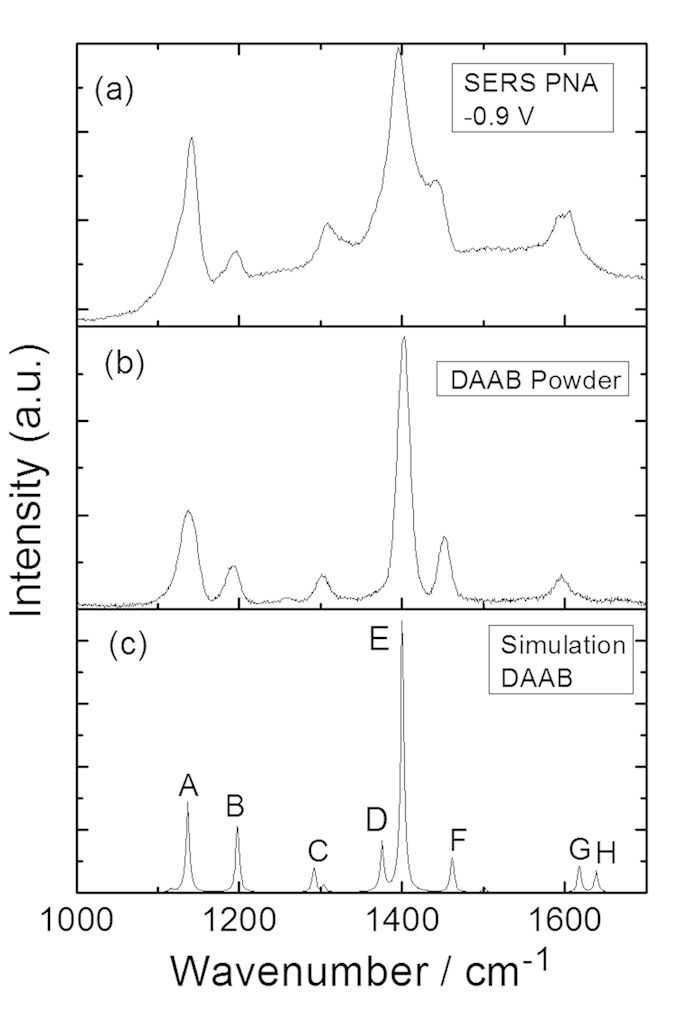
Comparison between experimental Raman spectra excited at 532 nm. (**a**) experimental SERS spectrum of PNA at −0.9 V, (**b**) experimental Raman spectrum of DAAB powder, and (**c**) the simulated Raman spectrum of DAAB.

**Figure 6:**
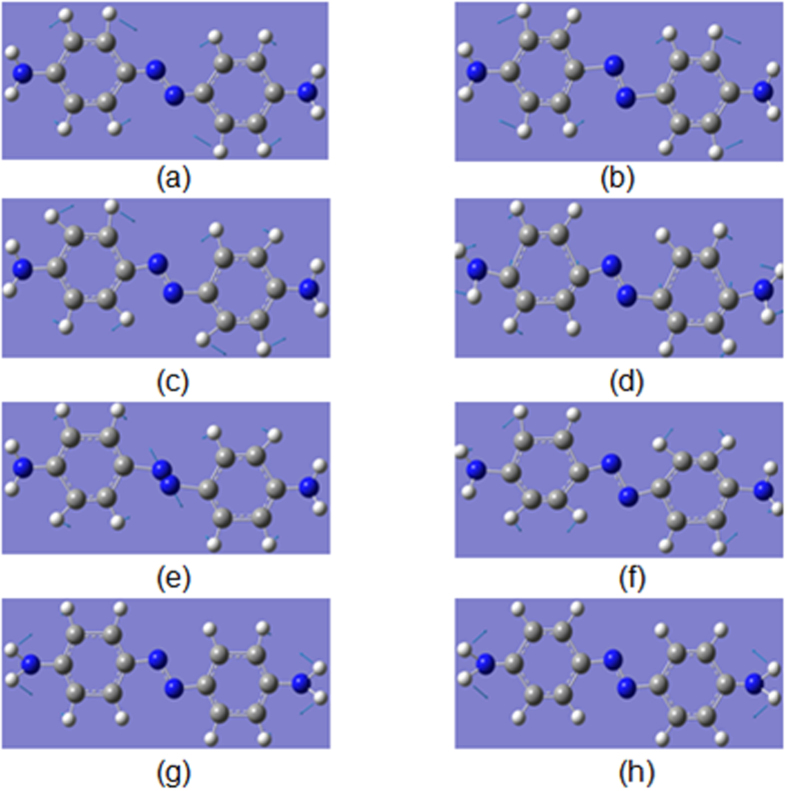
Calculated molecular Raman modes of DAAB. (**a–h**) different vibrational modes of DAAB.

To determine the reactions that occurred in Fig. 3(c), the Raman spectra of the DNAB, DAAB and NAAB powders were measured excited at 532 nm, as shown in Fig. 4. To provide more evidence, theoretical calculations of their Raman spectra were performed (see Fig. 4). Figure 4(a) shows the closest spectrum profile to that of Fig. 3c. For the convenience of comparing the results, the SERS spectrum of the PNA at −0.9 V, the Raman spectrum of the DAAB powder, and simulated Raman spectrum of the DAAB are presented together in Fig. 4. According to Fig. 5, it was concluded that the PNA was catalyzed to DAAB excited at 532 nm. The vibration Raman modes of the DAAB shown in Fig. 5c are illustrated in Fig. 6. Modes A and B are the C-N (−N = N−) stretching modes, modes C, D, E and F are the N = N stretching modes, mode G is the NH_2_ mode, and mode H is the NH_2_ scissoring and C-NH_2_ stretching mode.

**Figure 7:**
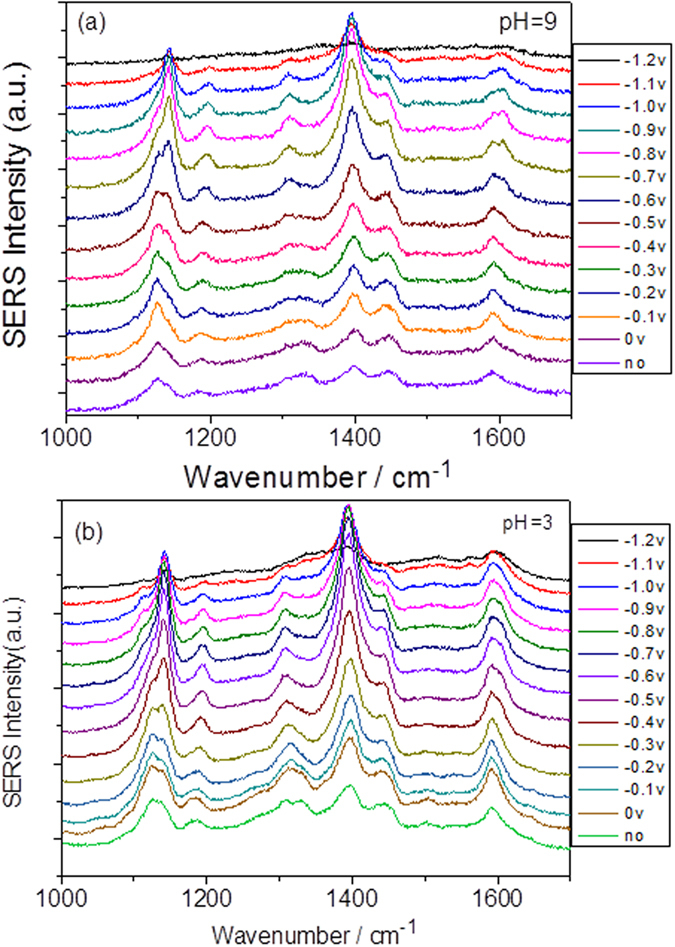
Potential dependent electrochemical SERS of PNA excited at 532 nm at different pH. (**a**) at pH = 9, and (**b**) pH = 3.

To verify the stability of the surface plasmon-assisted reduction of PNA to DAAB and the influences of the aqueous environment, two control experiments were performed. The electrode potential was scanned backwards from −1.2 V to 0 V, resulting in very stable SERS spectra (Fig. 3d). Next, the pH value of the aqueous environment (Fig. 7) was changed, which showed that the reduction is very stable and does not prevent the PNA from being catalyzed to DAAB on the roughened Ag substrate excited at 532 nm. Thus, our experimental results suggest a new method for synthesizing stable, new molecules using surface plasmon resonance.

Although PNA is not catalyzed to DNAB through the amine group or to NAAB through the nitro and amine groups, it is selectively catalyzed to DAAB through the nitro group. This clearly answers question presented in the title and demonstrates that plasmon-driven reduction occurs before plasmon-driven oxidation in aqueous environments. However, in an ambient environment, the plasmon-driven oxidation reaction via the amine group (−NH_2_) is favored over the plasmon-driven reduction reaction via the nitro group^1^,^17^. Thus, the plasmon-driven oxidation and reduction reactions can be controlled by the external environment. This phenomenon is explained by the abundant O_2_ on the substrate, which plays core role in the oxidation of the amine groups in ambient environments^15^. In contrast, amine oxidation is greatly restrained in an aqueous environment. The reduction of the nitro group is usually triggered by the hot electrons, which were generated from plasmon decay^5^,^25^, which would not be greatly affected by the reaction environment. Therefore, the reduction process of the nitro group is favored over the oxidation process of the amine group in an aqueous environment.

**Figure 8:**
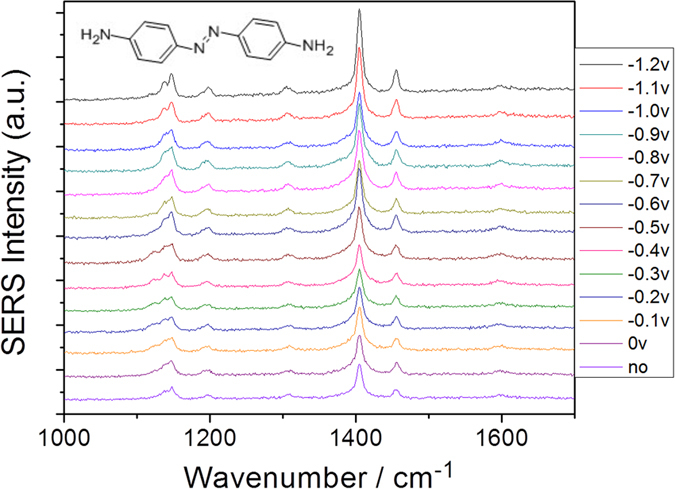
Potential dependent electrochemical SERS of DAAB excited at 532 nm.

**Figure 9:**
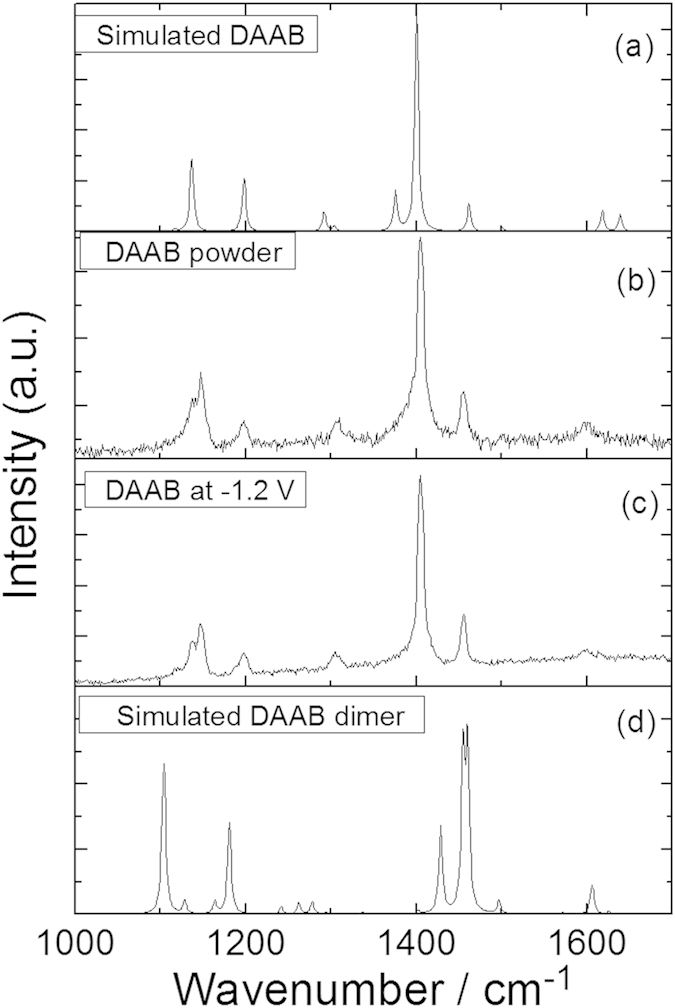
Comparisons between experimental Raman spectra excited at 532 nm. (**a**) Simulated Raman spectrum of DAAB, (**b**) Raman spectrum of DAAB powder, (**c**) experimental SERS spectrum of DAAB at −1.2 V, and (**d**) simulated Raman spectrum of DAAB dimer.

It is interesting that the DAAB is not further catalyzed to a new polymer (DAAB)n (where n stands for the unit number), with different DAAB units via two amine groups (−NH_2_) of DAAB in an aqueous environment. To explain this finding, we measured the potential-dependent electrochemical SERS of DAAB on the roughened Ag substrate in an aqueous environment excited at 532 nm (Fig. 8). It was found that the profile of the potential-dependent electrochemical SERS is the same as that of the DAAB powder in Fig. 4(a). Specifically, DAAB would not be catalyzed to a (DAAB)_n_ polymer via the amine groups by surface plasmon resonance in an aqueous environment. A comparison of the calculated Raman spectra of DAAB in (Fig. 9a) and (DAAB)_2_ in Fig. 9(d), together with the SERS of DAAB at −1.2 V (Fig. 9c) and the Raman spectra of DAAB (Fig. 9b), provide further evidence that the DAAB in an aqueous environment cannot be further catalyzed to a DAAB polymer. This result could be interpreted as the prevention of DAAB polymerization into (DAAB)_n_ due to the newly formed azo group (N = N).

**Figure 10:**
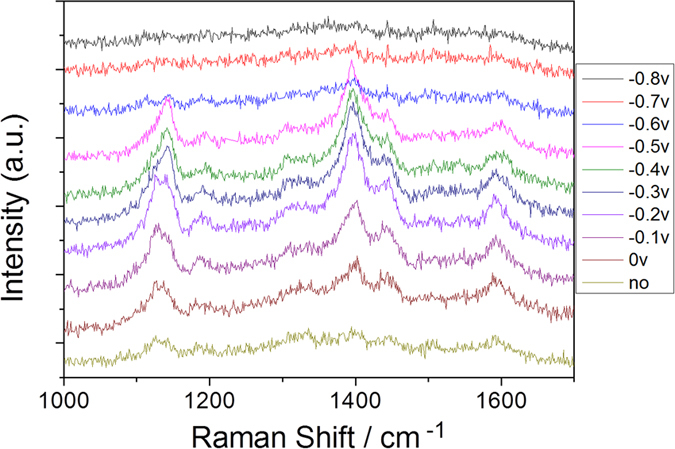
The experimental SERS spectrum of PNA at different potentials, excited at 532 nm. The sequence of measurements is from 0 V to −1.2 V.

To reveal the relationship between the measured reduction processes and surface plasmon resonance, two additional experimental works were performed. To demonstrate contribution the roughness of substrate for the plasmon-driven catalytic reaction, we also measured SERS of PNA (see Fig. 10) on the less roughened Ag substrate (Fig. 2b) excited at 532 nm and the stronger power of 1.5 mW. It is found the intensities of electrochemical SERS spectra on the less roughened substrate are weak at different potentials, though it the plasmon-driven chemical reaction can occur when potential is less −0.5 V. When the potentials are further increased from −0.6 V to −0.8 V, the Raman SERS spectra are too weak to be observed. So, the degree of roughness of substrate is a very important factor for plasmon-driven chemical reaction. The plasmon intensity is dependent on the degree of roughness of substrate. The larger roughness of Ag substrate, the stronger plasmon resonance is. So, this is an experimental evidence for the relationship between surface plasmon resonance and catalytic reaction.

**Figure 11:**
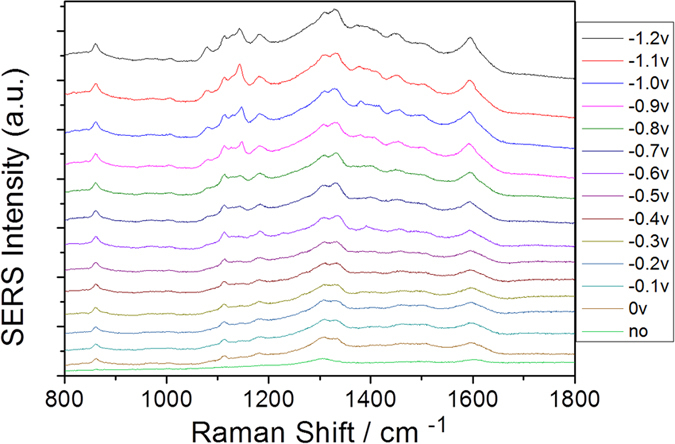
The experimental electrochemical SERS spectrum of PNA at different voltages excited at 785 nm. The sequence of measurements is from 0 V to −1.2 V.

We, furthermore, measured the electrochemical SERS of PNA (see Fig. 11) on the roughened Ag substrate at different voltages excited at 785 nm, where the laser intensity is 3.0 mW. The sequence of measurements is from 0 V to −1.2 V. It is found that there are no any chemical reactions, even under the help of external electric voltages. The reason is that the peak of surface plasmon resonance is far from 785 nm^13^. So, even stronger intensity of laser, the weak plasmon intensity excited at 785 nm can not drive catalytic reactions. This is the second experimental evidence for the relationship between surface plasmon resonance and catalytic reaction.

**Figure 12:**
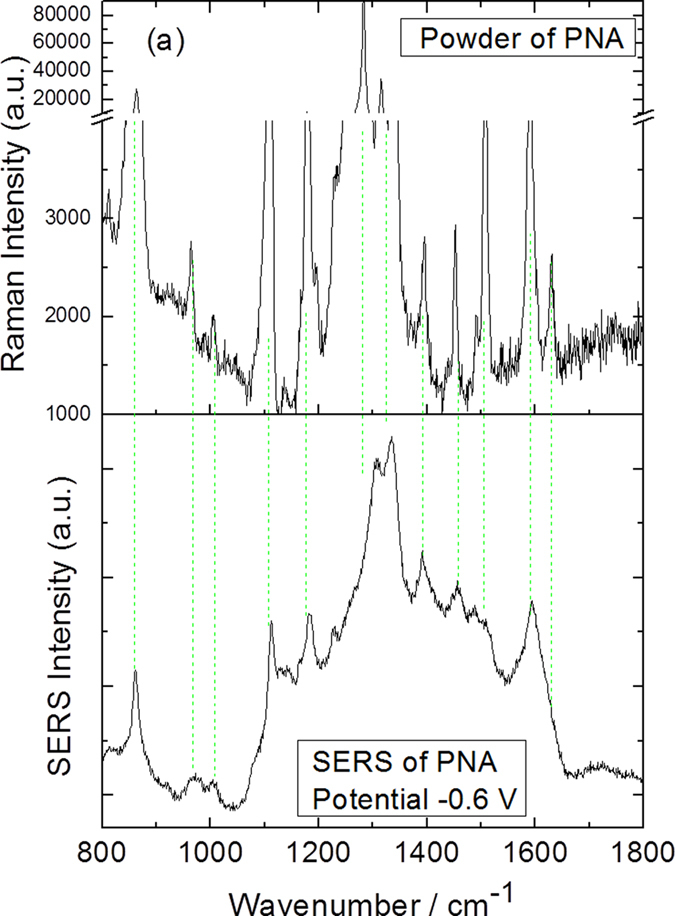
(**a**) The Raman spectrum of PNA power, and (**b**) the experimental electrochemical SERS spectrum of PNA at −0.6 V excited at 785 nm.

Though, there is no any chemical reaction for PNA on roughened Ag substrate at different potentials, excited at 785 nm, it is a good way to ascertain the SERS of the reactant of PNA, see the comparisons between normal Raman spectra of PNA with the electrochemical SERS spectrum at −0.6 V (see Fig. 12). Note that, the SERS spectra of the reactant of PNA excited at 532 nm (in Fig. 3a,b) can not be observed, due to the strong plasmon intensity. It is also the advantages of electrochemical SERS for ascertaining the SERS of the reactant of PNA, due to the weak surface plasmon resonance, excited at 785 nm.

The priority of the plasmon-driven oxidation and the reduction reactions in an aqueous environment was experimentally and theoretically investigated, using electrochemical SERS spectroscopy and density functional theory. The electrochemical SERS spectra of PNA (with both nitro (−NO_2_) and amine (−NH_2_) groups) and theoretical simulations revealed that PNA is selectively catalyzed to DAAB through the plasmon-assisted dimerization of the nitro (−NO_2_) group into an azo group in an aqueous environment. This indicates that the plasmon-driven reduction of the nitro (−NO_2_) group clearly occurs before the oxidation of the amine (−NH_2_) group in an aqueous environment. Two additional experimental measurements were performed to reveal the relationship between the measured reduction processes and surface plasmon resonance.

## Method

The PNA, NAAB and DAAB were purchased from Aldrich Chemical Co., Sigma Co. and Alfa Co., respectively. DNAB was synthesized by Beijing Kaida Co.

The SEM images of the Ag substrates were obtained using a Hitachi S-4800 microscope. The Raman spectra of the PNA, DAAB, NAAB and DNAB powders were measured using microprobe Raman system RH13325 spectrophotometer.

The Ag electrode (a single-crystal silver rod of 99.99% purity) was polished with emery paper and then was carefully cleaned with the Milli-Q water in the ultrasonic bath. And then, the Ag electrode was put into the electrochemical cell, in which the solution of 0.1 M Na_2_SO_4_ was used for roughening the Ag electrode. The double potential steps were used to roughen the surface of Ag electrodes, by applying the voltage of +0.25 V for 8 s, and then applying the voltage of −0.35 V. The roughening treatments were performed for enhancing Raman intensity. Lastly, the electrode was put into the electrochemical cell containing the solution of 0.1 M Na_2_SO_4_ with 0.02 M PNA.

Their Raman spectra were measured using the microprobe Raman system RH13325 spectrophotometer. The voltages of working electrode were controlled by the electrochemical instrument (CHI619B). The samples were excited with 532 nm and 785 nm lasers with an effective power of 0.3 mW, respectively where the 50× objective was used. The theoretical simulations of these molecular Raman spectra and their vibrational modes were calculated using with density functional theory (DFT)^26^, the 6-31G(d) basis set, and the pw91pw91 functional^27^. The calculated Raman spectra were scaled according to ref. 28. All the calculations were done with Gaussian 09 software.

## Additional Information

**How to cite this article**: Cui, L. *et al.* Selective plasmon-driven catalysis for para-nitroaniline in aqueous environments. *Sci. Rep.* **6**, 20458; doi: 10.1038/srep20458 (2016).

## Figures and Tables

**Figure 1:**
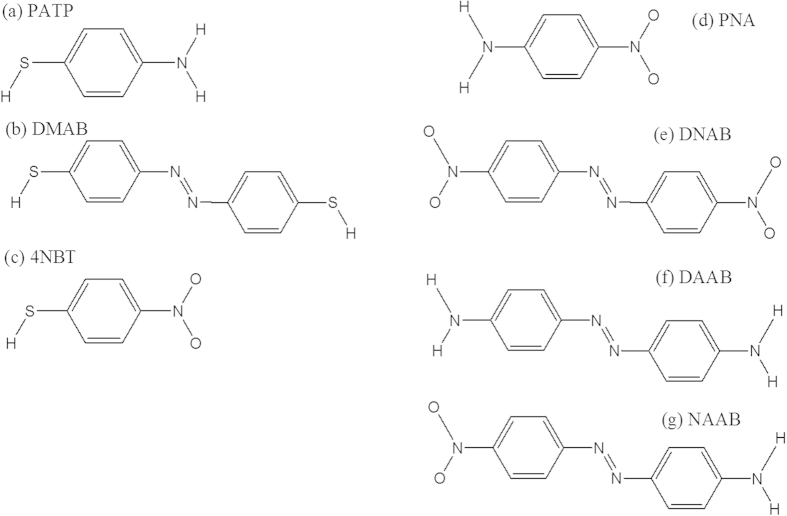
Molecular structures. (**a**) PATP, (**b**) DMAB, (**c**) 4NBT, (**d**) PNA, (**e**) DNAB, (**f**) DAAB and (**g**) NAAB.
